# Antibody Responses and Reactogenicity of a Heterologous, Full-Dose Messenger RNA-1273 Booster in Heavily SARS-CoV-2–Exposed CoronaVac-Vaccinated Health-Care Workers in Indonesia: A Real-World Observational Study

**DOI:** 10.4269/ajtmh.22-0256

**Published:** 2022-11-30

**Authors:** Robert Sinto, Dwi Utomo, Suwarti, Erni J. Nelwan, Henry Surendra, Cindy Natasha, Fransiska, Deborah Theresia, Adella Faiqa Ranitria, Decy Subekti, Nunung Nuraeni, Winahyu Handayani, Mutia Rahardjani, J. Kevin Baird, Susanna Dunachie, Anuraj H. Shankar, Raph L. Hamers

**Affiliations:** ^1^ Faculty of Medicine, Universitas Indonesia, Jakarta, Indonesia;; ^2^ Cipto Mangukusumo National Hospital, Jakarta, Indonesia;; ^3^ St Carolus Hospital, Jakarta, Indonesia;; ^4^ Centre for Tropical Medicine and Global Health, Nuffield Department of Medicine, University of Oxford, Oxford, United Kingdom;; ^5^ Pasar Minggu Hospital, Jakarta, Indonesia;; ^6^ Oxford University Clinical Research Unit Indonesia, Faculty of Medicine Universitas Indonesia, Jakarta, Indonesia;; ^7^ Infectious Disease and Immunology Research Cluster, Indonesian Medical Education and Research Institute, Jakarta, Indonesia;; ^8^ Mahidol-Oxford Tropical Medicine Research Unit, Mahidol University, Bangkok, Thailand

## Abstract

Real-world data on heterologous boosting with messenger RNA (mRNA)-1273 (Moderna) after inactivated COVID-19 vaccination are limited. We report mRNA-1273 boosting in heavily SARS-CoV-2–exposed Indonesian health-care workers who received a two-dose CoronaVac 6 months prior. Between August and November 2021, we measured SARS-CoV-2 spike-specific IgG binding antibody (Bab) titers in all 304 participants, and neutralizing antibody titers in a random subset of 71 participants, on stored paired serum samples taken before and 28 days after a full-dose (100-μg) mRNA-1273 booster. At the time of the mRNA-1273 boost, 107 participants (35.2%) were not previously infected (naive vaccinated), 42 (13.8%) were infected before CoronaVac (infected vaccinated), and 155 (51.0%) were infected after CoronaVac (mostly during the Delta wave; vaccinated infected). At time of the mRNA-1273 boost, neutralizing antibodies could still be detected in 83% of participants (59 of 71) overall, 60% of naive-vaccinated participants (15 of 25), 95.7% of vaccinated-infected participants (22 of 23), and 95.7% of infected vaccinated participants (22 of 23). After the mRNA-1273 boost, 100% of participants (71 of 71) had neutralizing antibody activity, with increases in median Bab and neutralizing antibody serum titers of 9.3- and 27.0-fold overall, 89.1- and 2,803.4-fold in naive-vaccinated participants, 15.9- and 19.9-fold in infected-vaccinated participants, and 2.2- and 18.4-fold in vaccinated-infected participants. In the multivariable analysis, Bab titers after the mRNA-1273 boost were greatest in individuals who had a previous virus breakthrough post-CoronaVac, and when a longer time period (> 4 months) had elapsed since the most recent prior “spike antigen exposure” (either second CoronaVac or virus breakthrough). Overall, adverse reactions were mild and short-lived. In conclusion, a full-dose mRNA-1273 booster after CoronaVac was well tolerated and immunogenic after 28 days, including in those with very low antibody levels.

## INTRODUCTION

Vaccination is a key global strategy to control the COVID-19 pandemic. To date, eight COVID-19 vaccines have received an Emergency Use Listing by the WHO,^1^ most of which require a two-dose regimen. Accumulating evidence shows a progressive increase in breakthrough infections over time since the second dose, which is associated with diminishing humoral immunity.^2^^–^^5^ Neutralization and vaccine effectiveness after two-dose vaccine schedules are particularly reduced for the recent Omicron variant (B.1.1.529),^6^ but with significant restoration after a third vaccine booster dose.^7^^–^^11^ Recent trials suggested that heterologous (or “mix and match”) virus-vectored or messenger RNA (mRNA) booster strategies were more immunogenic than a homologous schedule,^12^^–^^16^ albeit with increased reactogenicity in some combinations.^17^

CoronaVac (SinoVac Life Sciences Co. Ltd., Beijing, China), an inactivated whole-virus vaccine that received WHO Emergency Use Listing on June 1, 2021,^18^ is among the most widely administered COVID-19 vaccines worldwide, with more than 2 billion doses in 54 countries as of March 2022. Vaccine efficacy against symptomatic illness after two-dose CoronaVac was 50.5% to 83.5% in randomized trials.^18^^–^^20^ Vaccine effectiveness against hospitalization, intensive care unit admission, and COVID-19–related death was reported at 87.5%, 90.3%, and 86.3%, respectively, based on real-world data from Chile.^21^ However, recent studies reported CoronaVac to have lower immunogenicity than virus vector or mRNA vaccines,^22^^,^^23^ a pronounced decrease of neutralizing antibody titers within a few months,^23^^–^^25^ and reduced effectiveness in the elderly.^26^^,^^27^

CoronaVac has been the predominant vaccine rolled out in Indonesia’s countrywide mass vaccination campaign, which has seen the second highest number of COVID-19 cases (5,910,000) and deaths (153,000) in Asia (as of March 15, 2022).^28^ Frequent breakthrough infections during the second, Delta-driven wave in July 2021 prompted the Ministry of Health to offer a heterologous booster of full-dose (100-μg) mRNA-1273 vaccine (Moderna Inc., Cambridge, MA) to frontline health-care workers. Recent studies in individuals with no history of laboratory-confirmed COVID-19 found that an mRNA booster of BNT162b2 (Pfizer-BioNTech) after primary vaccination with CoronaVac was well tolerated and induced elevated, virus-specific antibody levels and potent neutralization activity, including against the Omicron variant, albeit to a lesser extent.^8^^–^^11^ However, real-world data are limited on mRNA-1273 as the booster as well as on “hybrid immunity” induced by previous SARS-CoV-2 infection.^29^

We therefore established the Indonesia Vaccine Immunity & Infection Evaluation (INVITE) study, which included an assessment of a full-dose (100-μg) mRNA-1273 booster dose given 6 months after two-dose CoronaVac among heavily SARS-CoV-2–exposed health-care workers in Jakarta, Indonesia. We documented the antispike (anti-S) IgG binding antibody (Bab) and neutralizing antibody (Nab) responses and reactogenicity of this booster, and examined the effects of previous SARS-CoV-2 infections occurring before or after primary CoronaVac vaccination.

## MATERIALS AND METHODS

### Design and population.

The INVITE study is a longitudinal observational cohort that includes health-care workers (doctors, nurses, and clinical laboratory analysts) who are 18 years or older at St Carolus and Pasar Minggu hospitals in Jakarta. We enrolled participants consecutively on the day they received their government-provided 100-μg mRNA-1273 booster dose at their own hospital, between August 5 and October 15, 2021. Eligible staff for the booster were those who had completed their two-dose primary vaccination at least 6 months prior, had no known recent (< 3 months) SARS-CoV-2 infection, and had no other contraindication for vaccination. Our analysis included all study participants for whom a paired pre- and postbooster serum sample was available. All participants provided written informed consent.

### Procedures.

Basic demographic and clinical data were captured on an online case report form. Solicited signs of local or systemic reactogenicity during the 7 days after the booster dose were recorded in a daily patient diary and, after study physician verification, were graded for severity (mild, moderate, severe, or disrupting activities of daily living [ADLs]). Previous COVID-19 was defined as a documented polymerase chain reaction–confirmed SARS-CoV-2 infection, and participants were thus categorized as not previously SARS-CoV 2-infected (naïve vaccinated), SARS-CoV-2–infected before the CoronaVac primary vaccination (infected vaccinated), or infected after the CoronaVac primary vaccination (vaccinated infected, also known as Delta breakthrough). Venous blood samples were drawn on the day of the booster dose before administration and 28 (+ 10) days thereafter (Supplemental Figure 1).

Serum was stored at –80°C and titers of IgG Babs against the SARS-CoV-2 spike receptor-binding domain were determined using the chemiluminescent microparticle immunoassay SARS-CoV-2 IgG II Quant assay (Abbott Laboratories, Abbott Park, IL) on the Architect i2000sr platform in accordance with the manufacturer’s instructions, and expressed in WHO International Standard binding antibody units (BAU) per milliliter using the manufacturer’s conversion factor (1 BAU/mL = 0.142 × arbitrary units/milliliter) and seropositivity defined as ≥ 7.1 BAU/mL.^30^ Samples with results greater than the upper limit of quantification were tested again after dilution. To validate the Bab measurements, we also measured Nabs using the SARS-CoV-2 Surrogate Virus Neutralization Test (cPass; GenScript, Piscataway, NJ) according to the manufacturer’s instructions, converted to WHO international units per milliliter for SARS-CoV-2 neutralization assays,^31^ in a random subset of the three participant groups. The cutoff value for Nabs was defined as 28 IU/mL or 30% signal inhibition.^31^^,^^32^

### Statistical analysis.

Paired comparisons of Bab and Nab titers before and after the mRNA-1273 booster dose were performed using the Wilcoxon matched pairs signed-rank test. Correlations of log_10_ titers were expressed using the Spearman coefficient (*r*_s_). Multivariable ordinal (proportional odds) logistic regression was performed to assess factors associated with pre- and postboost log_10_-transformed Bab titers (categorized in quartiles). Multivariable logistic regression was performed to assess factors associated with occurrence of any severe or disrupting adverse reactions. The independent variables included in the analysis were age, gender, previous SARS-CoV-2 infection, timing of previous SARS-CoV-2 infection before or after CoronaVac primary vaccination, any comorbidity (cardiovascular disease including hypertension, obesity [body mass index > 30 kg/m^2^], diabetes mellitus, and asthma), preboost Bab titer, time interval between the first and second CoronaVac dose, and time interval between the second CoronaVac and mRNA-1273 booster dose. A two-sided *P* < 0.05 was considered significant. Statistical analyses were performed with Stata/IC version 15.1 (StataCorp, College Station, TX).

## RESULTS

### Participant characteristics.

There were 304 of 353 participants (86.1%) with complete pre- and post-booster sample pairs who were included in the analysis. The median age was 31 years (interquartile range [IQR], 27–44 years; range, 21–59 years), 235 (77.3%) were women, and 61 (20.1%) had one or more comorbidities, including obesity, cardiovascular disease, diabetes mellitus, and asthma (Table 1). One hundred seven participants (35.2%) were naïve vaccinated, 42 (13.8%) were infected vaccinated, and 155 (51.0%) were vaccinated infected. The mRNA-1273 booster was given at a median of 190 days (IQR, 165–232 days) after the second CoronaVac dose.

**Table 1:** Participants characteristics

Characteristic	Total (*N* = 304)	Naive vaccinated (*n* = 107)	Infected vaccinated (*n* = 42)	Vaccinated infected (*n* = 155)
Age, years; median (IQR)	31 (27–44)	30 (26–43)	32 (28–46)	31 (27–45)
21–29, *n *(%)	134 (44.1)	52 (48.6)	12 (28.6)	70 (45.2)
30–39, *n *(%)	68 (22.4)	21 (19.6)	18 (42.9)	29 (18.7)
40–49, *n *(%)	57 (18.8)	23 (21.5)	4 (9.5)	30 (19.3)
50–59, *n *(%)	45 (14.8)	11 (10.3)	8 (19.0)	26 (16.8)
Gender				
Women, *n *(%)	235 (77.3)	83 (77.6)	30 (71.4)	122 (78.7)
Men, *n *(%)	69 (22.7)	24 (22.4)	12 (28.6)	33 (21.3)
No. of previous SARS-CoV-2 infections, *n *(%)				
0	107 (35.2)	107 (100.0)	0 (0.0)	0 (0.0)
1	176 (57.9)	0 (0.0)	27 (64.3)	149 (96.1)
2	21 (6.9)	0 (0.0)	15 (35.7)	6 (3.9)
Comorbidities, *n *(%)				
No	243 (79.9)	88 (82.2)	34 (81.0)	121 (78.1)
Yes	61 (20.1)	19 (17.8)	8 (19.0)	34 (21.9)
Obesity	39 (12.9)	13 (12.2)	6 (14.3)	20 (12.9)
Cardiovascular disease (including hypertension)	20 (6.6)	6 (5.6)	2 (4.8)	12 (7.7)
Asthma	7 (2.3)	2 (1.9)	1 (2.4)	4 (2.6)
Diabetes mellitus	4 (1.3)	2 (1.9)	1 (2.4)	1 (0.7)
Data before mRNA-1273 booster dose (day 0)				
Interval between first and second CoronaVac dose, days; median (IQR)	14 (14–17)	14 (14–17)	28 (14–31)	14 (14–14)
Interval between second CoronaVac dose and mRNA-1273 booster, days; median (IQR)	190 (165–232)	177 (151–190)	137 (94–153)	229 (196–241)
Interval between the most recent spike antigen exposure* and mRNA-1273 booster, days; median (IQR)	104 (89–171)	177 (151–190)	111 (92–147)	92 (79–101)
Bab titer, BAU/mL; median (IQR)	250 (32–1.389)	32 (15–84)	125 (42–760)	963 (292–2,116)
Bab seropositivity,† *n* (%)	293 (96.4)	97 (90.7)	42 (100.0)	154 (99.4)
Data after mRNA-1273 booster dose (day 28)				
Interval between mRNA-1273 booster and day 28 Bab measurement, days; median (IQR)	34 (31–36)	35 (34–39)	35 (31–43)	31 (29–34)
Bab titer day 28, BAU/mL; median (IQR)	2,313 (1,226–4,324)	2,834 (1,228–4,687)	1,986 (1,153–4,078)	2,136 (1,299–3,855)
Bab seropositivity,† *n* %	304 (100)	107 (100)	42 (100)	155 (100)

Bab = binding antibody; BAU = binding antibody units (according to the WHO International Standard); IQR = interquartile range; mRNA = messenger RNA.

* Defined as either CoronaVac second dose or breakthrough infection, whichever occurred most recently.

† Binding antibody seropositivity was defined as ≥ 7.1 BAU/mL.

### Antibody titers before and after mRNA-1273 booster.

**Figure 1.**
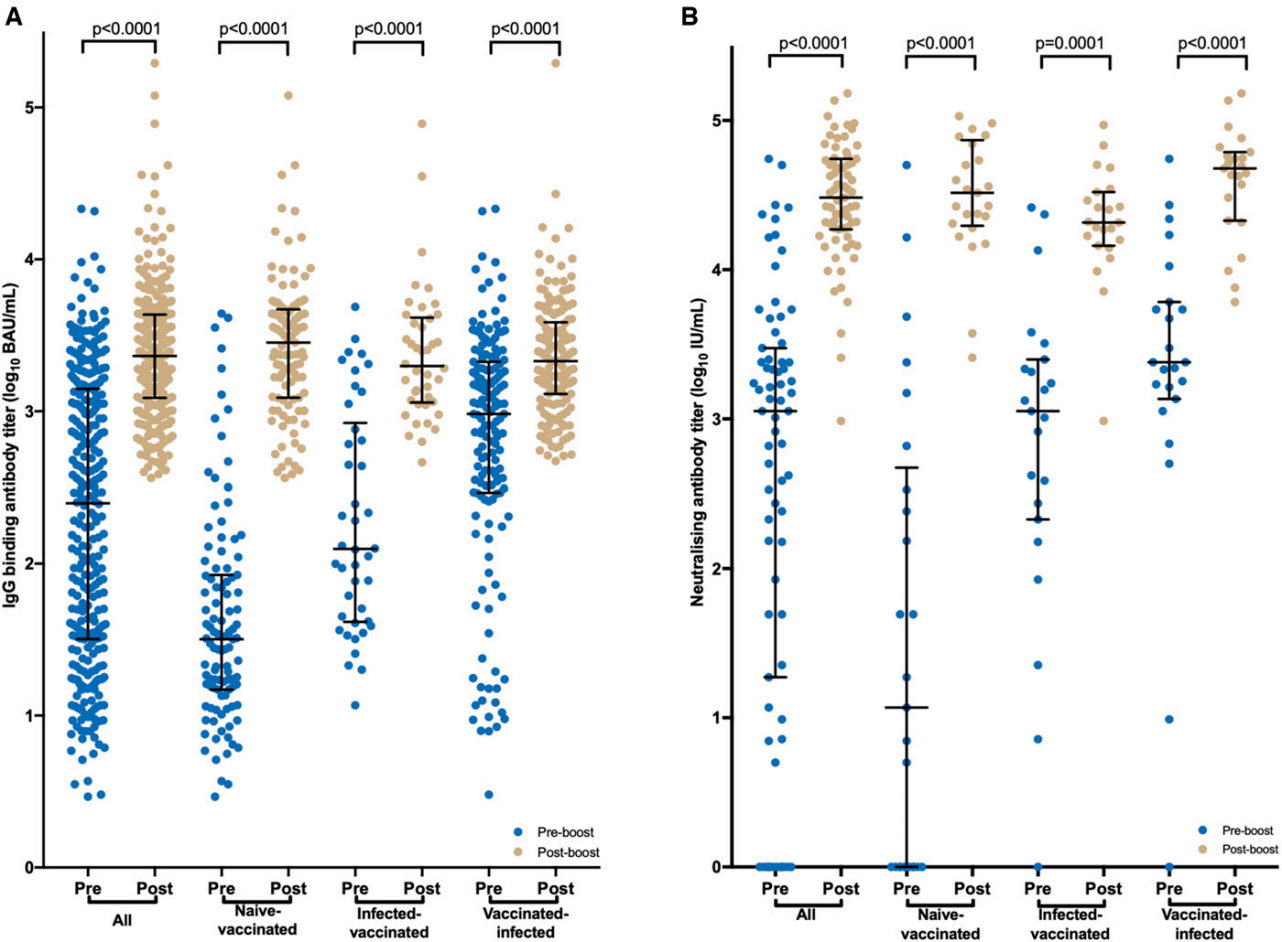
Antispike binding and neutralizing antibody titers before and after messenger RNA-1273 booster dot plot showing before and after messenger RNA-1273 booster binding antibody (**A**) and neutralizing (**B**) titers overall and by previous SARS-CoV-2 infection. IgG titers shown as BAU per milliliter (according to the WHO International Standard). BAU = binding antibody units.

The proportion of Bab-seropositive participants increased from 96.4% (*n* = 293) before receiving the mRNA-1273 booster (which was ∼6 months after receiving CoronaVac, as described earlier) to 100% (*n* = 304) after boosting (Table 1). After receiving the mRNA-1273 booster, the median Bab titer increased 9.3-fold overall, from 250 BAU/mL (IQR, 32–1,389 BAU/mL) before to 2,313 BAU/mL (IQR, 1226–4324 BAU/mL; *P *< 0.001) after. Naive-vaccinated participants had a 89.1-fold increase (from 31.8 BAU/mL [IQR, 14.8–84.3 BAU/mL] to 2,833.6 BAU/mL [IQR, 1,227.6–4,686.7]), infected-vaccinated participants had a 15.9-fold increase (from 124.9 BAU/mL [IQR, 41.7–760.3 BAU/mL] to 1,985.5 BAU/mL [IQR, 1,153.0–4,077.5 BAU/mL]), and vaccinated-infected participants had a 2.2-fold increase (from 962.6 BAU/mL [IQR, 292.0–2,115.5 BAU/mL] to 2,136.4 BAU/mL [IQR, 1,299.3–3854.6 BAU/mL]; *P* < 0.0001 each) (Figure 1, Supplemental Figure 2). Supplemental Figure 2 and Supplemental Table 1 summarize pre- and post-mRNA-1273 boost titers by previous SARS-CoV-2 infection, age, gender, and presence of any comorbidity.

At the time of the mRNA-1273 boost, Nab activity could still be detected in 83% of participants overall (59 of 71), in 60% of the naive-vaccinated (15 of 25), 95.7% of the vaccinated-infected (22 of 23), and 95.7% of the infected-vaccinated (22 of 23). After receiving the mRNA-1273 boost, 100% (71 of 71) had Nab activity, and the Nab titer increased 27.0-fold overall—from 1,127.6 IU/mL (IQR, 18.7–2,986.1 IU/mL) before to 30,400.0 IU/mL (IQR, 18,640.0–55,310.8 IU/mL; *P* < 0.0001) after (*N* = 71). Naive-vaccinated participants (*n* = 25) had a 2,803.4-fold increase (11.7 IU/mL [IQR, 0–336.8 IU/mL] to 32,800.0 IU/mL [20,392.0–69,574.2 IU/mL]; *P* < 0.0001), infected-vaccinated participants (*n* = 23) had a 19.9-fold increase (2,400.0 IU/mL [IQR, 1,359.4–6,073.0 IU/mL] to 47,760.0 IU/mL [IQR, 21,340.0–61,400.0 IU/mL]; *P* = 0.0001), and vaccinated-infected participants (*n* = 23) had a 18.4-fold increase (1,127.6 IU/mL [IQR, 213.5–2,501.0 IU/mL] to 20,720.0 IU/mL [IQR, 14,480–33,120 IU/mL]; *P* < 0.0001) (Figure 1).

### Correlations between antibody titers before and after mRNA-1273 booster.

**Figure 2.**
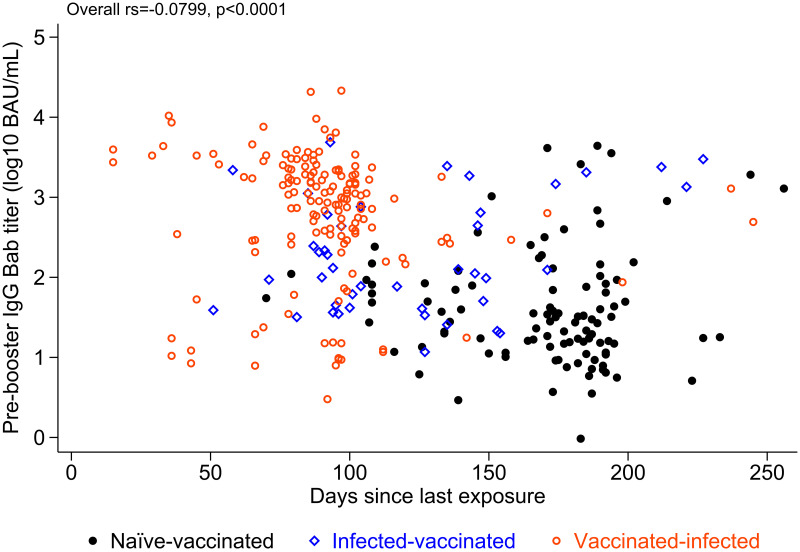
Antispike binding antibody (Bab) titers before messenger RNA (mRNA)-1273 booster and time since most recent “spike antigen exposure.” Scatterplot of antispike Bab titers before mRNA-1273 booster and time since most recent spike antigen exposure (either by the CoronaVac second dose or SARS-CoV-2 infection, whichever was the most recent). Pre-mRNA-1273 boost Bab titers were higher overall in previously infected-vaccinated than in naive-vaccinated participants. Pre-mRNA-1273 boost Bab titers correlated inversely with the time since the most recent documented spike antigen exposure. Participants with a breakthrough infection post-CoronaVac (vaccinated-infected) had the shortest time since most recent spike antigen exposure, and had a higher pre-mRNA-1273 boost Bab titer than those infected before CoronaVac (infected-vaccinated) and naive-vaccinated participants. *BAU = binding antibody units*.

**Figure 3.**
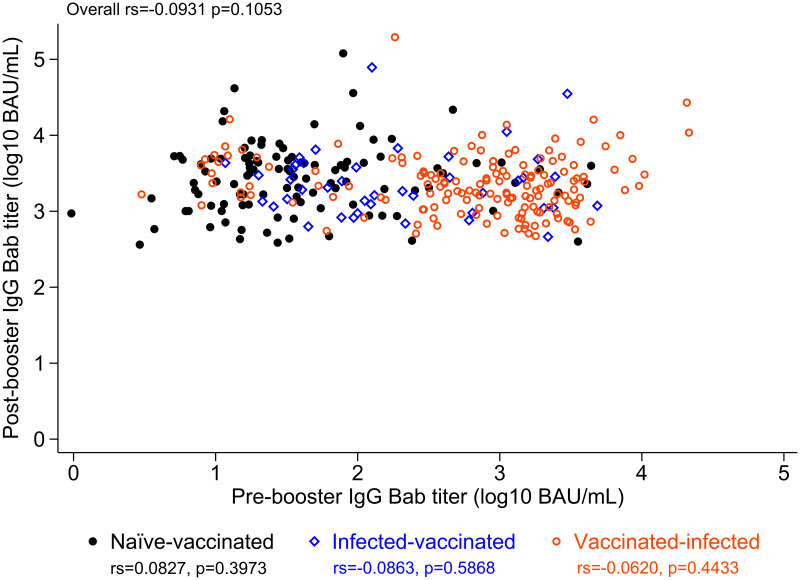
Antispike binding antibody (Bab) titers before and after messenger RNA (mRNA)-1273 booster. Scatterplot of antispike Bab titers before and after mRNA-1273 booster. All participants reached a substantial post-mRNA-1273 boost Bab titer, ranging between 2,570 and 1,380,384 BAU per milliliter (2.73 log_10_ range), and this was regardless of the pre-mRNA-1273 boost Bab titer, previous infection, or the timing of the previous infection (before or after a two-dose CoronaVac vaccination). Participants with a breakthrough infection post-CoronaVac (vaccinated-infected) had a higher pre-mRNA-1273 boost Bab titer than those infected before CoronaVac (infected-vaccinated) and the naive-vaccinated participants. Post-mRNA-1273 boost Bab titers were in the same range for all three subgroups. BAU = binding antibody units.

Pre-mRNA-1273 boost Bab titers varied widely, from 0.97 to 21,466 BAU/mL (4.35 log_10_ range), and were greater in those previously infected (Figures 2 and 3). More recent spike antigen exposure, either by the CoronaVac second dose or breakthrough infection (whichever was most recent), correlated with greater pre-mRNA-1273 boost Bab and Nab titers (Figures 1 and 2). Vaccinated-infected participants had the shortest time since most recent spike antigen exposure, and had a greater pre-mRNA-1273 boost Bab titer than either infected-vaccinated or naive-vaccinated participants (Figure 2). All participants reached substantial post-mRNA-1273 boost antibody titers, ranging between 364 and 195,452 BAU/mL (2.73 log_10_ range) for Babs and 968 and 432,880 IU/mL (2.65 log_10_ range) for Nabs (Figures 1 and 3, Supplemental Figure 2).

Pre-mRNA-1273 boost Bab titers correlated significantly with Nab in naive-vaccinated participants (r_s_ = 0.8813, *P* < 0.0001) and infected-vaccinated participants (r_s_ = 0.4398, *P* = 0.0357), but not in vaccinated-infected participants (r_s_ = 0.0089, *P* = 0.9679). Post-mRNA-1273 boost Bab titers correlated, although insignificantly, with Nabs in naive-vaccinated participants (r_s_ = 0.3531, *P* = 0.0834), but did not correlate in infected-vaccinated participants (r_s_ = –0.1156, *P* = 0.5993) and vaccinated-infected participants (r_s_ = –0.1294, *P* = 0.5561) (Supplemental Figure 3).

### Factors associated with pre- and postbooster Bab titers

In multivariable analysis, previous SARS-CoV-2 infection either before or after the CoronaVac primary vaccination was strongly associated with a greater preboost Bab titer (*P* = 0.001 and *P* < 0.0001, respectively) and, as expected, pre-mRNA-1273 boost Bab titers were lower when a longer period of time (> 3 months) had elapsed since the most recent prior spike antigen exposure (either the second CoronaVac dose or a breakthrough infection) compared with a more recent exposure.

Post-mRNA-1273 boost Bab titers were greater in participants who had a previous SARS-CoV-2 infection after the CoronaVac primary vaccination (i.e., virus breakthrough), and when a longer period of time (> 4 months) had elapsed since the most recent prior spike antigen exposure (either the second CoronaVac dose or a breakthrough infection) compared with more recent exposure. Age, gender, comorbidity, and time interval between the first and second CoronaVac dose were not associated with the pre- or post-mRNA-1273 boost Bab titer; and pre-mRNA-1273 boost Bab titer and previous SARS-CoV-2 infection before CoronaVac were not associated with the post-mRNA-1273-boost Bab titer (Table 2).

**Table 2:** Regression analysis of pre- and post-messenger RNA-1273 boost log_10_ antispike binding antibody titers

Independent variable	Pre-mRNA-1273 boost Bab						Post-mRNA-1273 boost Bab					
	Univariable			Multivariable			Univariable			Multivariable		
	Beta	95% CI	*P* value	Beta	95% CI	*P *value	Beta	95% CI	*P* value	Beta	95% CI	*P *value
Age (per decade)	0.095	–0.094 to 0.283	0.325	0.019	–0.180 to 0.219	0.849	0.111	–0.079 to 0.301	0.255	–0.001	–0.208 to 0.205	0.989
Gender												
Men	0 (ref)	–	–	–	–	–	0 (ref)	–	–	–	–	–
Women	–0.230	–0.717 to 0.256	0.354	–0.355	–0.862 to 0.153	0.171	–0.015	–0.500 to 0.470	0.951	0.000	–0.495 to 0.495	1.000
Comorbidity												
Cardiovascular diseases	0.418	–0.475 to 1.310	0.359	–	–	–	1.020	0.158 to 1.882	0.020	0.863	–0.064 to 1.789	0.068
Diabetes	–0.510	–0.251 to 1.489	0.617	–	–	–	2.360	0.133 to 4.587	0.038	2.106	–0.238 to 4.449	0.078
Obesity	0.270	–0.344 to 0.885	0.388	–	–	–	–0.163	–0.763 to 0.436	0.593	–	–	–
Asthma	–0.990	–2.316 to 0.337	0.144	–	–	–	0.377	–1.026 to 1.780	0.599	–	–	–
Previous SARS-CoV-2 infection, timing												
Naive vaccinated	0 (ref)	–	–	–	–	–	0 (ref)	–	–	–	–	–
Infected vaccinated	1.489	0.834 to 2.144	< 0.0001	1.316	0.545 to 2.086	0.001	–0.380	–1.024 to 0.264	0.248	0.299	–0.478 to 1.075	0.451
Vaccinated infected	2.782	2.234 to 3.331	< 0.0001	2.464	1.709 to 3.219	< 0.0001	–0.333	–0.778 to 0.113	0.143	0.829	0.014 to 1.644	0.046
Interval between first and second dose, day	–0.007	–0.018 to 0.006	0.296	–	–	–	0.001	–0.013 to 0.015	0.883	–	–	–
Interval between most recent spike antigen exposure* and mRNA-1273 booster, months												
< 3	0 (ref)	–	–	–	–	–	0 (ref)	–	–	–	–	–
3–4	–1.031	–1.591 to –0.471	< 0.0001	–0.899	–1.465 to –0.333	0.002	0.152	–0.381 to 0.686	0.576	0.272	–0.280 to 0.823	0.334
4–5	–2.207	–2.980 to –1.434	< 0.0001	–1.008	–1.856 to –0.160	0.020	0.567	–0.157 to 1.290	0.125	1.041	0.162 to 1.921	0.020
5–6	–2.707	–3.487 to –1.927	< 0.0001	–1.114	–2.034 to –0.193	0.018	0.544	–0.152 to 1.241	0.126	1.041	0.107 to 1.975	0.029
> 6	–2.603	–3.300 to –1.907	< 0.0001	–0.840	–1.710 to 0.031	0.059	0.932	0.310 to 1.553	0.003	1.576	0.661 to 2.491	0.001
Preboost Bab titer	–	–	–	–	–	–	–0.191	–0.413 to 0.032	0.094	–0.135	–0.411 to 0.140	0.336

Bab = binding antibody; mRNA = messenger RNA; ref (reference).

The table shows the results of univariable and multivariable ordinal regression analyses with preboost and postboost Bab titer as the dependent variables (classified in quartiles).

* Defined as either CoronaVac second dose or breakthrough infection, whichever occurred most recently.

### Reactogenicity of mRNA-1273 booster.

**Figure 4.**
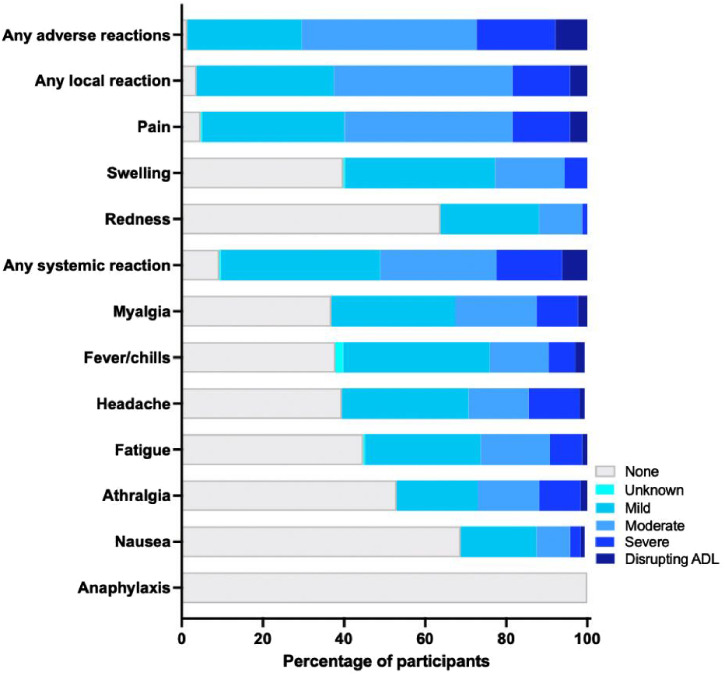
Adverse reactions within 7 days after receiving the messenger RNA-1273 booster dose. The bar chart shows solicited adverse reactions that were verified and graded for severity by the study physician. Severity grading: 1, mild (does not or interferes minimally with usual social and functional activities); 2, moderate (interferes with usual social and functional activities); 3, severe (causes inability to perform usual social and functional activities); and 4, disrupting/impairing (disrupts/impairs ADL). No participants required hospitalization or died. ADL = activities of daily living.

The 304 participants reported a total of 300 adverse reactions within 7 days after receiving the mRNA-1273 booster. The percentage of participants with any solicited adverse reaction was 98.7% (*n *= 300), 96.4% (*n *= 293) for any local reaction (injection site pain and swelling most frequent), and 90.8% (*n * =276) for any systemic reaction (myalgia, fever/chills, and headache being most frequent). Most solicited local and systemic adverse reactions were mild (*n *= 86, 28.3%) or moderate (*n *= 131, 43.1%), whereas severe (*n *= 59, 19.4%) or those disrupting ADLs (*n *= 24, 7.9%) were less common (Figure 4, Supplemental Table 2). All adverse reactions were short-lived and none required hospitalization. Severe or ADL-disrupting adverse reactions were associated with a longer interval between the second dose and the mRNA-1273 booster (OR, 1.37/month increase; IQR, 1.09–1.72; *P* = 0.001) and were inversely associated with older age (OR, 0.71/age decade increase; IQR, 0.54–0.92; *P* = 0.011). Gender, comorbidity, previous SARS-CoV-2 infection, time interval between the first and second dose, and pre- and postboost Bab titers were not associated with any adverse reactions (Supplemental Table 3).

## DISCUSSION

This real-world study in Indonesian health-care workers who received two-dose CoronaVac primary vaccination 6 months prior found that humoral immunity diminished substantially over time, as reported previously.^23^^–^^25^ As expected, this was more pronounced in those who were not previously infected with SARS-CoV-2 than in those who had hybrid immunity resulting from previous infection. The heterologous mRNA-1273 vaccine booster dose increased Bab and Nab titers significantly in all tested individuals, including those who had undetectable antibody levels before the booster. In adjusted multivariable analysis, Bab titers after receiving the mRNA-1273 booster were found to be greatest in individuals who had a history of a virus breakthrough after the primary CoronaVac vaccination, and in those for whom a longer time period (> 4 months) had elapsed since the most recent prior spike antigen exposure (either the second CoronaVac dose or a virus breakthrough) compared with more recent exposure. This finding of a longer interval between vaccine doses being beneficial for antibody production builds on existing literature for doses 1 and 2 of Oxford/AstraZeneca and mRNA vaccines.^33^^–^^35^ Our observations confirm intense SARS-CoV-2 exposure among frontline health workers in Jakarta during the Delta wave in July 2021.^36^ Our findings concur with the notion that Delta breakthrough infections postvaccination are associated with subsequent more potent and durable immune responses than those seen in individuals who were infected only or received only two doses of the COVID-19 vaccine.^37^

From an immunological perspective, we noted a lower pre-mRNA-1273 booster correlation between Bab and Nab titers in the vaccinated-infected group, wherein the most recent exposure to the spike protein was from natural infection rather than vaccination. This finding is consistent with natural infection generating diverse anti-S Bab to non-neutralizing epitopes—a notion consistent with a recent study^38^ in which plasma from individuals who had been infected and subsequently received mRNA vaccination could better neutralize an engineered SARS-CoV-2 polymutant spike protein or diverse sarbecovirus spike proteins. Moreover, we surmise the observed low post-mRNA-1273 booster Bab and Nab titer correlations could be attributed to antibody production ceiling effects, as reported previously^39^ for the association between anti-S1 IgG titers and the inhibition of the binding between S1 and angiotensin converting enzyme 2 after the second mRNA vaccine dose.

The high-dose heterologous mRNA-1273 booster was generally well tolerated, with common reports of injection site pain and swelling; as well as muscle pain, fever/chills, and headache; and 7.9% experienced a short-lived impairment in performing ADLs. The mRNA-1273 booster was better tolerated with increasing age and for a shorter time period since the second CoronaVac dose. These findings largely concur with Moderna’s phase II/III trial of the high-dose (100-μg) booster dose, which was generally safe and well tolerated, with only slightly more frequent adverse reactions compared with the 50-μg dose.^40^

Our study findings add to current evidence on the use of mRNA booster vaccines in three main ways. First, it established that heterologous mRNA vaccine boosting with mRNA-1273 (Moderna) after CoronaVac is immunogenic and well tolerated in a highly virus-exposed population reflecting real-world conditions.^29^ This concurs with other recent trials in which heterologous boosting with the other widely used mRNA vaccine BNT162b2 (Pfizer-BioNTech) after CoronaVac demonstrated better immunogenicity than a homologous CoronaVac booster against wild-type and Delta and Omicron variants.^8^^–^^11^ Heterologous boosting has been shown to result in an enhanced quantitative profile of antibody and T-cell responses, and expands the ability to recognize multiple regions of spike compared with a homologous booster.^41^^,^^42^ Second, our study evaluated a 100-μg full dose of the mRNA-1273 booster rather than the authorized 50-μg booster dose. On December 12,, 2021, Moderna announced^40^ preliminary data that a mRNA-1273 booster of 50 μg increased Nabs 37-fold, and that of 100 μg increased Nabs 83-fold, compared with preboost levels, in person without previous infection. Third, the findings contribute to informing policy on flexible options in deploying COVID-19 vaccines in mix-and-match schedules, with particular relevance for countries that are largely dependent on inactivated vaccines. As policymakers in several countries have started implementing third or periodic boosting to protect the most vulnerable populations, and mitigate health-care and economic impacts, real-world data will be critical to guide decisions regarding when, which populations, and which boosters should be administered.

There are some limitations to our study. First, we did not assess anti-N IgG, Nabs against Delta and Omicron variants of concern, or cellular immunity. Although accumulating evidence suggests that the anti-S IgG Bab and Nab responses correlate with protection against infection,^43^^,^^44^ it is important to recognize there is not yet an established or well-defined correlate of long-term vaccine protection,^45^ and protection against hospitalization and death is likely to involve Bab and T-cell responses, which is supported by global data showing decreased risk of severe disease after Omicron infection compared with previous waves in well-vaccinated populations.^46^ Second, we could not fully confirm all who had been infected by SARS-CoV-2 because routine surveillance may have been incomplete, and the use of anti-N IgG as a proxy for previous infection was not valid because of the initial immunization with a whole-virus inactivated vaccine. Third, the sample size and follow-up period were insufficient to identify less common or late adverse events after booster vaccination, and the immunogenicity data were limited to immune responses through study day 28. Fourth, only immunological data were collected and therefore this investigation lacks information regarding the effectiveness of a heterologous mRNA-1273 booster vaccination, in particular against infection with the emergent BA.1 and BA.2 subvariants, and future variants. Last, demographics of the volunteers were not representative of the Indonesian population, and elderly individuals were not represented.

In conclusion, our real-world data indicate that, after a primary course of two-dose CoronaVac inactivated vaccine, a heterologous, full-dose mRNA-1273 booster was immunogenic after 28 days of follow-up and was well tolerated among heavily SARS-CoV-2–exposed health-care workers, even in those with very low preboost antibody titers. Further trials on clinical end points of optimized booster doses and variant-specific or multivalent vaccines in response to decreased susceptibility to neutralization of emerging SARS-CoV-2 variants of concern are warranted.^47^

## Supplemental files


Supplemental materials
