# Supplementary material for: Antibody Responses and Reactogenicity of a Heterologous, Full-Dose Messenger RNA-1273 Booster in Heavily SARS-CoV-2–Exposed CoronaVac-Vaccinated Health-Care Workers in Indonesia: A Real-World Observational Study
Source: Am J Trop Med Hyg. 2022 Nov 30;108(1):115–23. doi: 10.4269/ajtmh.22-0256 (PMC9833072; doi:10.4269/ajtmh.22-0256)
Supplement: Supplementary file 1 [file tpmd220256.SD1.pdf]

Figure S1. Study participant timeline

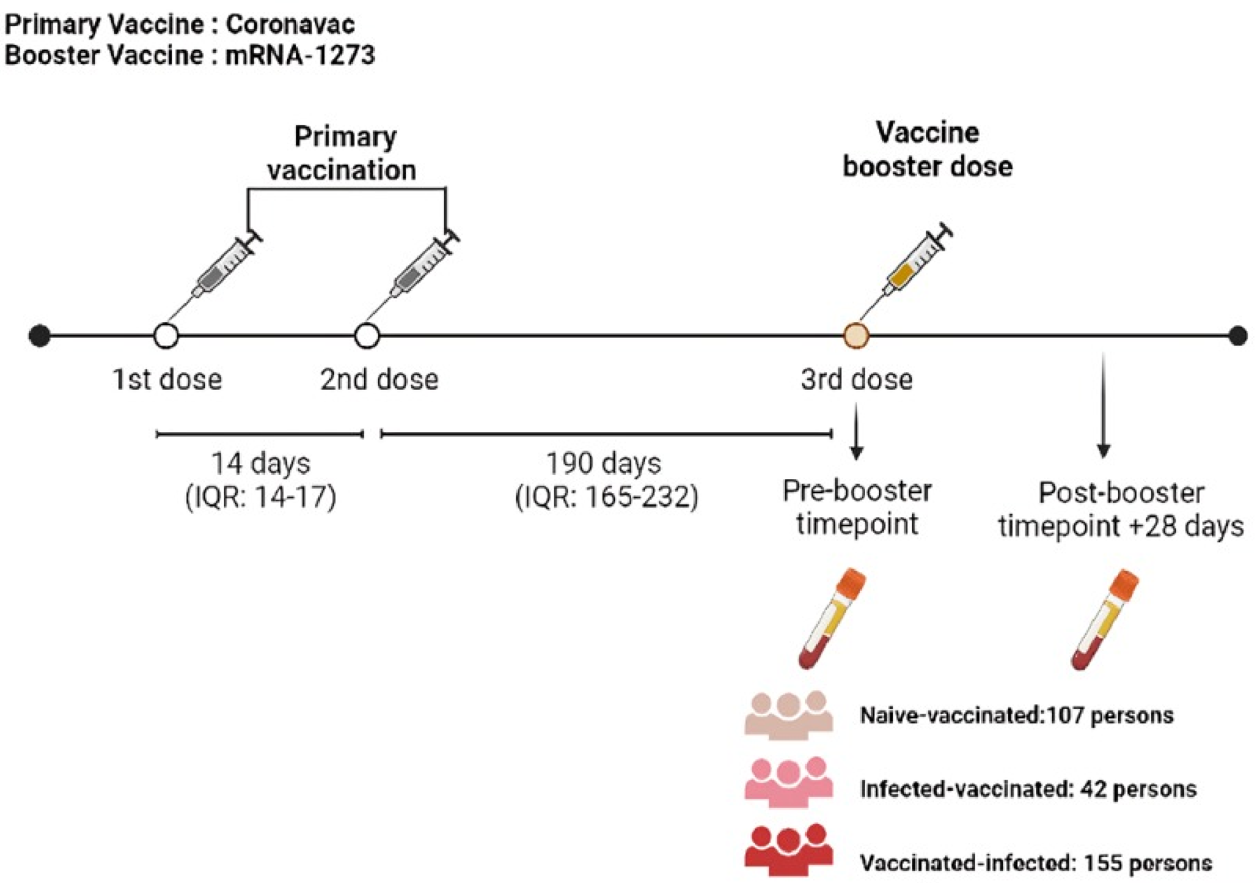

Figure S2. Anti-spike binding antibody titers before and after mRNA-1273 booster

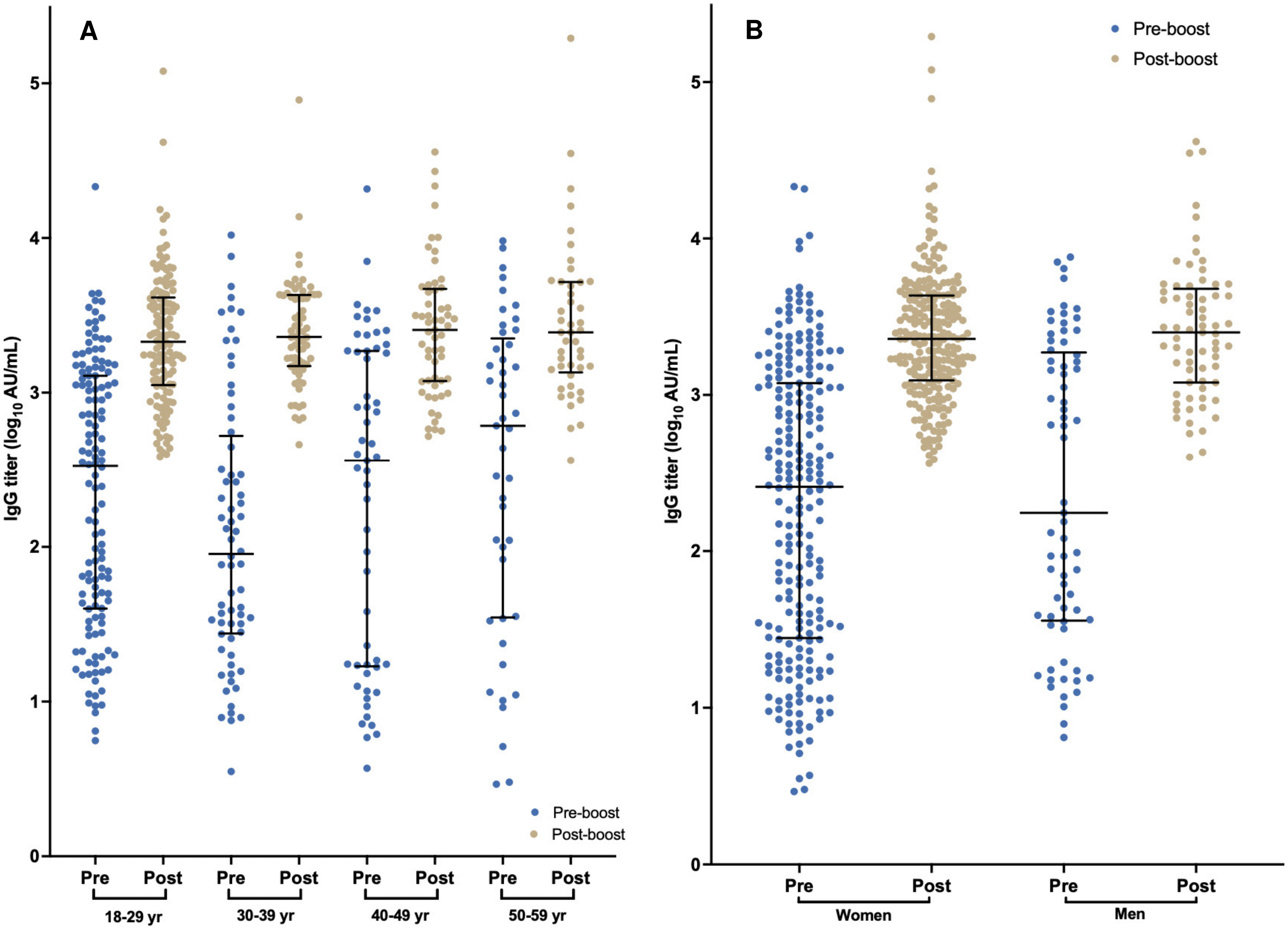

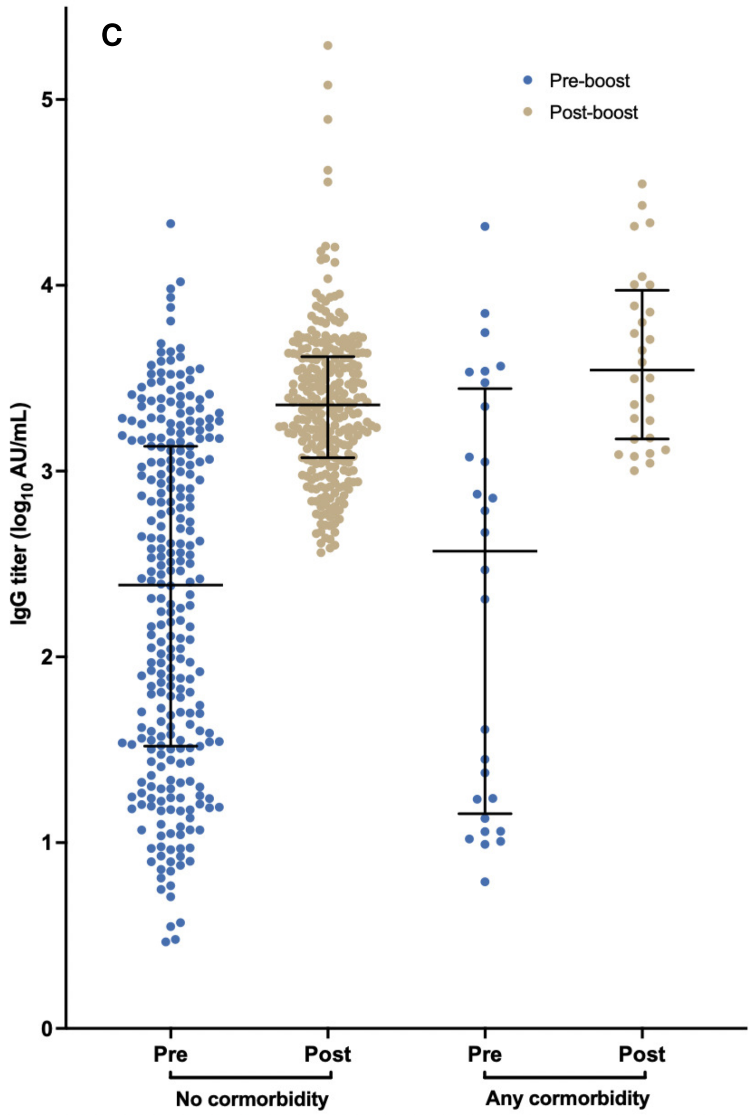

Panels (A-C) show dot plots of pre and post-mRNA-1273-booster binding antibody titers, by (A) age groups, (B) sex, (C) comorbidity.

IgG binding antibody titers shown as binding antibody units (BAU)/mL (according to WHO International Standard).

Figure S3. Correlations between binding and neutralizing antibody titers before and after mRNA-1273 booster

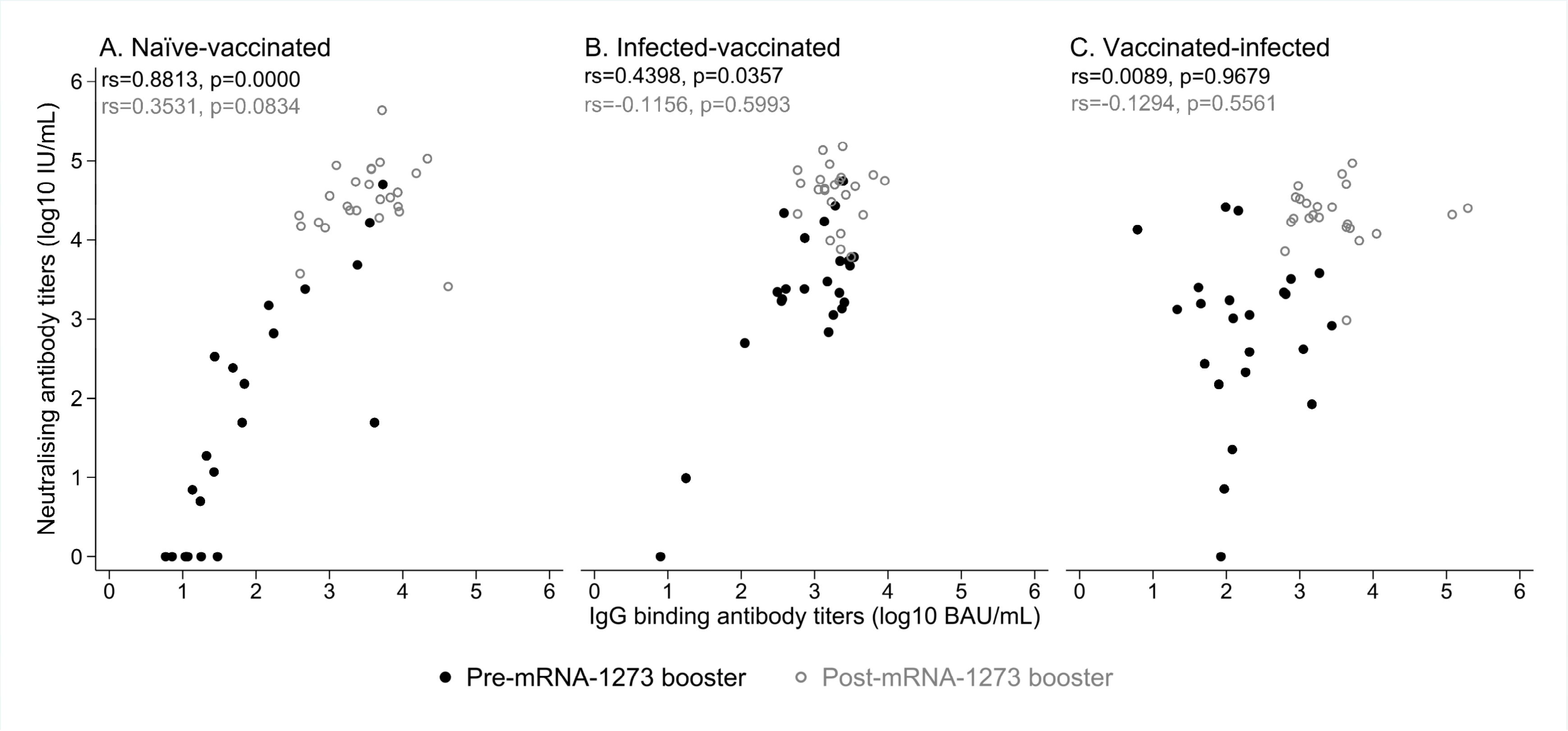

Scatter plot showing correlations between pre and post-mRNA-1273-booster binding and neutralizing antibody titers, overall and by previous SARS-CoV-2 infection. IgG titers shown as binding antibody units (BAU)/mL (according to WHO International Standard). Neutralizing antibodies shown as IU/mL.

Table S1. Anti-spike titers before and Day 28 after mRNA-1273 booster

	Median pre-mRNA-1273-booster (IQR)	Median post- mRNA-1273-booster (IQR)	Fold change	p-value
Overall	249.8 (31.9-1389.2)	2312.8 (1225.8-4324.3)	9.3	<0.0001
Age				
21-29	334.9 (39.9-1285.1)	2132.8 (1128.2-4066.2)	6.4	<0.0001
30-39	90.2 (27.7-500.4)	2292.2 (1500.2-4271.4)	25.0	<0.0001
40-49	363.1 (17.1-1856.7)	2543.9 (1197.1-4594.4)	7.0	<0.0001
50-59	611.0 (35.5-2048.7)	2455.2 (1410.2-5177.3)	2.8	0.0001
Sex				
Women	257.1 (27.9-1191.1)	2278.4 (1238.2-4315.4)	8.9	<0.0001
Men	175.2 (36.4-1804.3)	2510.6 (1202.7-4570.3)	14.3	<0.0001
Previous SARS-CoV2 infection - N episodes				
0	31.8 (14.8-84.3)	2833.6 (1227.6-4686.7)	89.1	<0.0001
1	808.5 (193.5-2049.1)	2163.0 (1301.4-4033.3)	2.7	<0.0001
2	257.1 (61.5-994.1)	1741.6 (823.6-2492.8)	6.8	0.0010
Previous SARS-CoV-2 infection - Timing				
Naïve-vaccinated	31.8 (14.8-84.3)	2833.6 (1227.6-4686.7)	89.1	<0.0001
Infected-vaccinated	124.9 (41.7-760.3)	1985.5 (1153.0-4077.5)	15.9	<0.0001
Vaccinated-infected	962.6 (292.0-2115.5)	2136.4 (1299.3-3854.6)	2.2	<0.0001
Time interval between second dose and mRNA-1273 booster				
Quartile 1 (66-165 days)	80.1 (34.3-211.6)	2369.6 (1202.0-4324.3)	24.6	<0.0001
Quartile 2 (165-190 days)	33.0 (15.5-294.0)	2943.0 (1482.5-4686.7)	94.6	<0.0001
Quartile 3 (190-232 days)	709.4 (49.6-1596.1)	2522.3 (1303.6-5305.1)	3.4	<0.0001
Quartile 4 (232-267 days)	1439.5 (533.8-2226.6)	1714.7 (1132.5-2764.0)	1.2	0.0037
Comorbidity				
0	243.9 (33.1-1359.9)	2269.9 (1180.7-4115.5)	9.3	<0.0001
≥1	380.9 (15.3-2608.7)	3510.3 (1494.9-8898.8)	9.2	<0.0001

Data are expressed as binding antibody units (BAU)/mL (according to WHO International Standard)

Table S2. Adverse reactions within seven days after receiving the mRNA-1273 booster dose

	**N (%)**	**Grade 1**	**Grade 2**	**Grade 3**	**Grade 4**	**Unknown**
Any adverse reactions	300 (98.68)	86 (28.29)	131 (43.09)	59 (19.41)	24 (7.89)	0 (0.00)
Any local reaction	293 (96.38)	103 (33.88)	134 (44.08)	43 (14.14)	13 (4.28)	0 (0.00)
Pain	290 (95.39)	107 (35.20)	126 (41.45)	43 (14.14)	13 (4.28)	1 (0.33
Swelling	183 (60.20)	113 (37.17)	52 (17.11)	17 (5.59)	0 (0.00)	1 (0.33)
Redness	110 (36.18)	74 (24.34)	32 (10.53)	4 (1.32)	0 (0.00)	0 (0.00)
Any systemic reaction	276 (90.79)	120 (39.47)	87 (28.62)	49 (16.12)	19 (6.25)	1 (0.33)
Myalgia	192 (63.16)	93 (30.59)	61 (20.07)	31 (10.20)	7 (2.30)	0 (0.00)
Fever/chills	189 (62.17)	110 (36.18)	44 (14.47)	20 (6.58)	7 (2.30)	6 (1.97)
Headache	184 (60.53)	95 (31.25)	45 (14.80)	38 (12.50)	4 (1.32)	0 (0.00)
Fatigue	168 (55.26)	87 (28.62)	52 (17.11)	24 (7.89)	4 (1.32)	1 (0.33)
Arthralgia	143 (47.04)	61 (20.07)	46 (15.13)	31 (10.20)	5 (1.64)	0 (0.00)
Nausea	95 (31.25)	57 (18.75)	25 (8.22)	8 (2.63)	3 (0.99)	0 (0.00)
Anaphylaxis	0 (0.0)	0 (0.00)	0 (0.00)	0 (0.00)	0 (0.00)	0 (0.00)

The table shows solicited adverse reactions, that were verified and graded for severity by the study physician. Severity grading: 1) mild (does nor or minimally interfere with usual social and functional activities); 2) moderate (interferes with usual social and functional activities); 3) severe (causing inability to perform usual social and functional activities); 4) disrupting/impairing activities of daily living (ADL). No participants required hospitalization or died.

Table S3. Factors associated with occurrence of severe or disrupting adverse reactions

	Univariable			Multivariable		
	**OR**	**95%CI**	**P**	**OR**	**95%CI**	**P**
Age, per decade increase	0.69	0.53-0.90	0.006	**0.71**	**0.54-0.92**	**0.011**
Sex						
Men	1 (ref)			1 (ref)		
Women	1.63	0.85-3.130	0.139	1.60	0.81-3.17	0.174
Comorbidities						
No	1 (ref)			-	-	-
Yes	0.88	0.36-2.15	0.774	-	-	-
Past COVID-19						
No	1 (ref)			1 (ref)		
Yes	1.73	0.99-3.03	0.053	1.20	0.59-2.44	0.619
Pre-mRNA-1273-booster binding antibody titer	1.43	1.08-1.89	0.013	0.73	0.33-1.61	0.431
Post-mRNA-1273-booster binding antibody titer	0.58	0.31-1.10	0.095	-	-	-
Change in binding antibody titers between before and after mRNA-1273 boost	0.69	0.53-0.89	0.004	0.65	0.33-1.30	0.225
Interval between first and second vaccination, per day increase	0.97	0.93-1.00	0.059	0.99	0.96-1.02	0.484
Interval between second vaccination and mRNA-1273 booster dose, per month increase	**1.51**	**1.25-1.83**	**<0.0001**	**1.37**	**1.09-1.72**	**0.007**

Table shows univariable and multivariable logistic regression with occurrence of any severe or ADL-disrupting adverse reaction (within seven days after receiving the mRNA-1273 booster dose) as the dependent variable.

Severe or disrupting adverse reactions were defined as causing inability to perform usual social and functional activities or disrupting/impairing activities of daily living (ADL), respectively.

Interval between first and second dose, post-mRNA-1273-booster binding antibody titer, and presence of any comorbidity were not associated with the occurrence of severe or disrupting adverse reaction.
